# Local administration of HMGB-1 promotes bone regeneration on the critical-sized mandibular defects in rabbits

**DOI:** 10.1038/s41598-021-88195-7

**Published:** 2021-04-26

**Authors:** Ahmed Monir, Taro Mukaibo, Abdel Basit M. Abd El-Aal, Tomotaka Nodai, Takashi Munemasa, Yusuke Kondo, Chihiro Masaki, Mahasen A. El-Shair, Kou Matsuo, Ryuji Hosokawa

**Affiliations:** 1 Division of Oral Reconstruction and Rehabilitation, Kyushu Dental University, Kitakyushu, Fukuoka Japan; 2 Department of Surgery, Anesthesiology, and Radiology, Faculty of Veterinary Medicine, Zagazig University, Sharkia, Egypt; 3 Division of Oral Pathology, Department of Health Promotion, Kyushu Dental University, Kitakyushu, Fukuoka Japan

**Keywords:** Experimental models of disease, Oral diseases, Molecular medicine

## Abstract

Reconstruction of a critical-sized osseous defect is challenging in maxillofacial surgery. Despite novel treatments and advances in supportive therapies, severe complications including infection, nonunion, and malunion can still occur. Here, we aimed to assess the use of a beta-tricalcium phosphate (β-TCP) scaffold loaded with high mobility group box-1 protein (HMGB-1) as a novel critical-sized bone defect treatment in rabbits. The study was performed on 15 specific pathogen-free New Zealand rabbits divided into three groups: Group A had an osseous defect filled with a β-TCP scaffold loaded with phosphate-buffered saline (PBS) (100 µL/scaffold), the defect in group B was filled with recombinant human bone morphogenetic protein 2 (rhBMP-2) (10 µg/100 µL), and the defect in group C was loaded with HMGB-1 (10 µg/100 µL). Micro-computed tomography (CT) examination demonstrated that group C (HMGB-1) showed the highest new bone volume ratio, with a mean value of 66.5%, followed by the group B (rhBMP-2) (31.0%), and group A (Control) (7.1%). Histological examination of the HMGB-1 treated group showed a vast area covered by lamellar and woven bone surrounding the β-TCP granule remnants. These results suggest that HMGB-1 could be an effective alternative molecule for bone regeneration in critical-sized mandibular bone defects.

## Introduction

The etiology of a critical-sized defect in the mandible includes accidental trauma, infection, and tumor resection^1^. A critical-size defect will neither heal spontaneously^2^, nor will it regenerate more than 10% of the lost bone during the patient’s lifetime^3^. Ideally, mandibular reconstruction aims to restore form and function as rapidly as possible^4^.

Autologous bone grafting is known as the gold standard approach for osseous regeneration^5,6^. However, it has significant limitations and drawbacks, including donor site morbidity and a finite amount of autologous bone that matches the structural and functional properties of the defective part. Moreover, additional complications, including hematoma, infection, increased operative time and bleeding, chronic donor site pain, and extra costs have been reported^7,8^. Homologous and heterologous bone grafts are widely used as alternatives, but carry the risks of disease transmission, variable resorption, and potential adverse autoimmune reactions^9^.

Because of these limitations and the expanding need for bone reconstruction, there is great interest in the use of bone substitutes and tissue engineering^10^. The tissue engineering concept in bone regeneration involves the utilization of growth factors, a scaffold material that mimics the microenvironment of bone tissue, and progenitor cells that can grow to form bone cells^11,12^.

Bone morphogenic proteins (BMPs) belong to the transforming growth factor β (TGF-β) superfamily. They were identified by Urist in 1965 for their ability to initiate ectopic bone formation^13–15^. Several studies have demonstrated the ability of BMPs to facilitate the repair of critical-sized mandibular defects with augmenting substitutes in animal models^9,16,17^. BMP-2 and BMP-7 are potent osteoblast differentiation factors with widespread therapeutic potential for targeting bone defects^18^. Many preclinical and human clinical studies have employed rhBMP-2 in the craniofacial skeleton^19–22^. However, variable outcomes have been reported regarding the use of rhBMP-2 in the craniomaxillofacial area since its approval by the Food and Drug Administration (FDA) in 2007^23^.

rhBMP-2 has been studied in combination with resorbable collagen^24,25^, collagen sponge with hydroxyapatite and β-tricalcium phosphate crystals^26^, and a poly d, l-lactic-co-glycolic-acid coated gelatin sponge^27^. Although rhBMP-2 is one of the most commonly used growth factors in tissue engineering strategies, some adverse clinical outcomes appear to be associated with its clinical use, including aberrant inflammatory responses, inadequate bone quality, ectopic bone formation, and a higher dose requirement^28,29^.

Recently, several studies have focused on high mobility group box 1 protein (HMGB-1). It is a non-histone, highly conserved DNA-binding nucleoprotein that is localized in the nucleus, but can be actively secreted extracellularly by various cells, or passively secreted by dead and necrotic cells^30,31^. HMGB-1, also known as “alarmin” was initially considered as a death mediator; an endogenous molecule released by dead or dying cells that alerts the innate immune system to tissue damage and the need for repair or apoptosis^32–34^.

Several reports have suggested that the anabolic properties of HMGB-1 during tissue regeneration can act as a critical mediator of systemic and local inflammation^30,32,35,36^. Regarding its bone bioactivity, the HMGB-1 receptor is expressed in mesenchymal stem cells (MSCs). Meng et al. demonstrated that HMGB-1 promotes the migration of MSCs and their differentiation along the osteoblastic pathway, indicating the role of HMGB-1 in bone restoration^37^. Wolf et al. showed human periodontal ligament cell proliferation, migration, and osteoblast differentiation using HMGB-1 treatment^38^. Moreover, previous reports have demonstrated that HMGB-1 can be released by bone cells, including osteoblasts, osteoclasts, Mlo-y4 osteocyte-like cells, and MC3T3-E1 osteoblast-like cells, and that this expression is increased by hypoxia, which resembles the bone fracture microenvironment^39^.

Li et al. found that HMGB-1 can promote a 2–3fold increase in the osteoblast migration rate through the Toll-like receptor (TLR)2, TLR4, and nuclear factor kappa B (NF-κB) signaling pathways without causing any cytotoxic effects^40^. A novel in vivo study showed that HMGB-1 promoted fracture healing when used locally in conjunction with a mesenchymal stem cell sheet^41^.

In this study, we evaluated the feasibility of using HMGB-1 as a sole bone-active molecule for the regeneration of critical-sized mandibular osseous defects in rabbits using radiographic and histological assessments.

## Results

### Clinical findings

All surgical interventions were successfully performed without any intraoperative complications. Minor postoperative surgical swelling was observed in five rabbits, which lasted for 3 days, and the rabbits spontaneously recovered without any specific interference. All cases showed a decrease in food intake and loss of appetite, which returned to normal within 3–5 days. All animals successfully completed the course of the study without signs of infection, debilitation, or mandibular loss of function.

### Radiographic examination

Periodic X-ray examination was performed on all animals every 4 weeks to evaluate the condition of the scaffold material and tissue reactions.

#### Group A (control)

**Figure 1:**
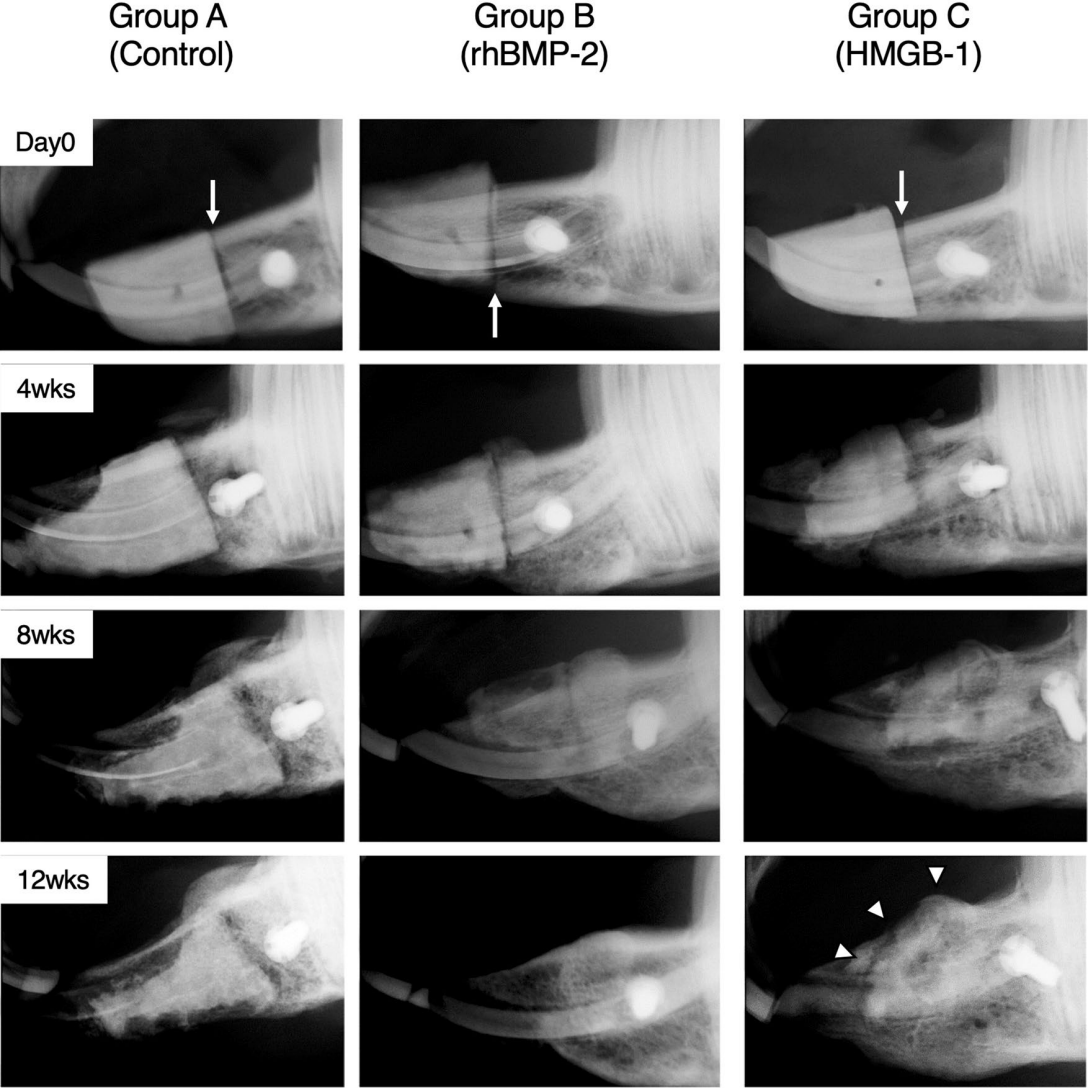
Periodical x-ray examination of the lateral view every 4 weeks. Periodic X-ray examination showed the different reactions of the scaffold and bone in response to each substance. On day 0, X-ray images of all groups showed a clear demarcation as a radiolucent line between the bone and scaffold material (white arrow). At 4 weeks postoperatively, the line started to fade in group C, whereas the line was still present in groups A and B. At 8 weeks postoperatively, group A still showed a clear line, while a significant change in shape and size was identified in groups B and C. At this stage, The radiolucent line was almost undetectable in group C. In the final week, 12 weeks postoperatively, the scaffold was still identifiable as a high radiodensity structure in group A. In contrast, the scaffold appeared to be absorbed in group B. Substantial bridging formation was observed in group C (white arrowheads).

Periodic X-ray images of group A (control) showed minimal to no bony reactions around the scaffold material. A demarcation line between the bone and the scaffold material could be recognized as a radiolucent line between the bone and the scaffold material. In the first month, no changes were identified in the scaffold material or the surrounding tissue. However, at 8 and 12 weeks postoperatively, a slight change in the radiodensity of the scaffold material and a minor tissue reaction were observed without visible new bone regrowth (Fig. 1).

#### Group B (rhBMP-2)

Group B (rhBMP-2) showed slight changes in the volume and shape of the scaffold material at 4 weeks postoperatively. However, no prominent tissue reaction or bridging formation was observed. After eight weeks, a major change in the scaffold material and bridging formation between the scaffold material and the adjacent bones was observed. By the end of the 12-week postoperative period, moderate bone reaction and new bone formation were identified, and the whole scaffold material was nearly resorbed (Fig. 1).

#### Group C (HMGB-1)

Group C (HMGB-1) showed early changes in the shape, volume, and density of the scaffold material at 4 weeks postoperatively. Following bridging formation between the scaffold material and the adjacent bone, the line of demarcation between the scaffold material and the adjacent bone started to fade at this stage. By the end of the 12-week postoperative period, a substantial radiodense structure surrounding the remnants of the scaffold material was observed in the X-ray images (Fig. 1).

### Micro-computed tomography (CT) examination

**Figure 2:**
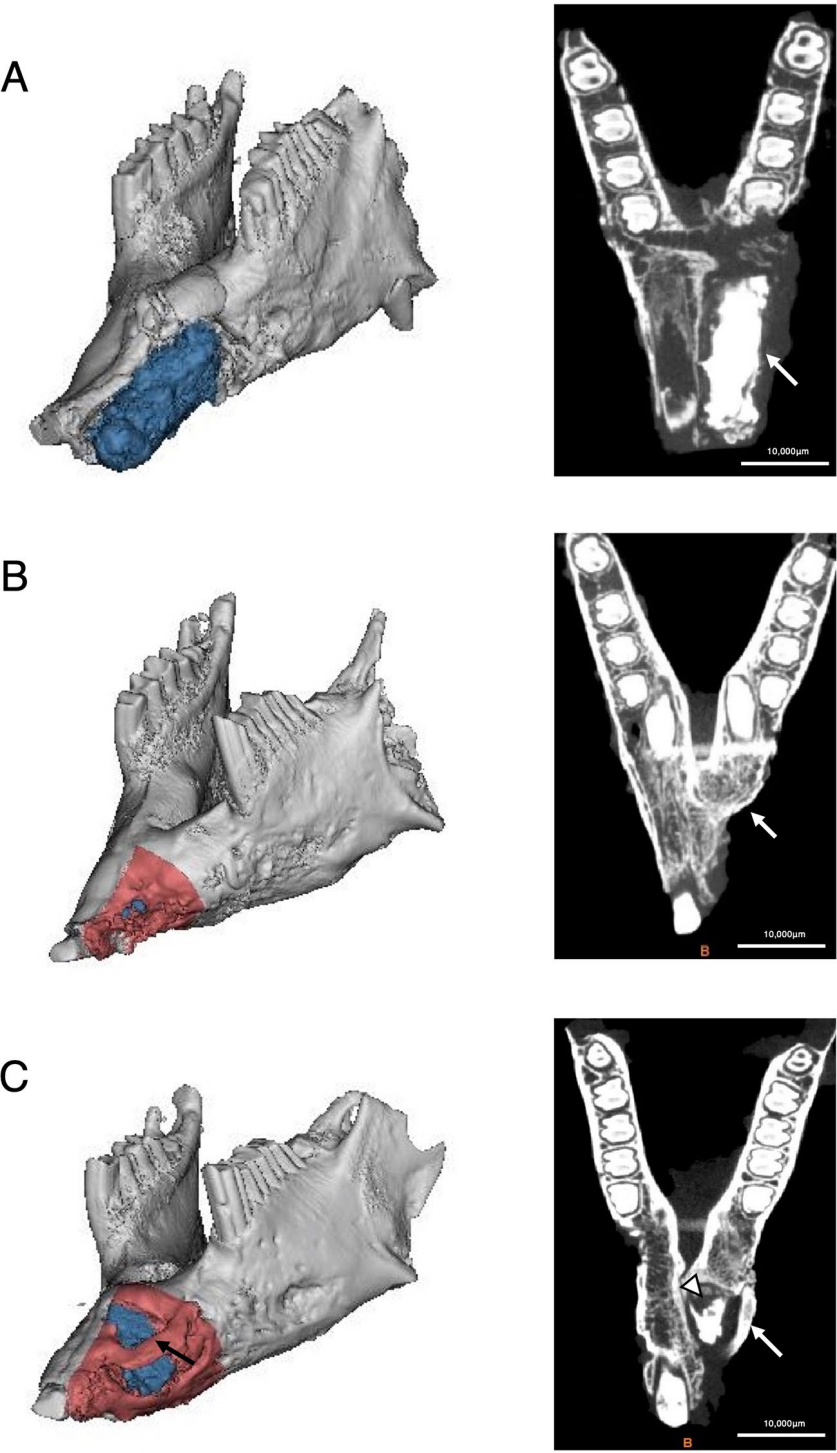
Micro-computed tomography (CT) assessment of regenerated bone formation. Micro-CT data and its three-dimensional (3-D) reconstructed images were used to differentiate new bone formation and remnant scaffold material at 12 weeks after the procedure. (**A**) In group A (control), an extensive remnant scaffold material (blue) is present in the left panel. In the axial section image, the scaffold is identified as a high radiodensity structure in the right panel (white arrow). (**B**) In group B (rhBMP-2), the scaffold is not detectable (left panel). No radiodensity structure corresponding to the scaffold is detectable in the axial section image (right panel). At the same time, minimal bone formation was identified (white arrow). (**C**) In group C (HMGB-1), extensive callus formation (black arrow) was found surrounding the remnant scaffold (left panel). The axial section image shows the bridging formation (white arrow) and the remnant scaffold being absorbed and replaced by a lower radiodensity structure (white arrowhead, right panel). The remnant scaffold is identified in blue and newly formed bone in red in the 3-D reconstructed images. The images in (**A**) were retrieved from specimen ID 18–3, (**B**) from 18–13, and (**C**) from 18–5. Materialise MIMICS software version 21.0 (https://www.materialise.com/en/medical/mimics-innovation-suite/mimics) was used for the analysis. Individual axial micro-CT images of specimens are presented in Supplementary Fig. S2.

CT images of group A (Control) showed a regenerated bone volume of 37.4 ± 16.8 mm^3^, while the remnant scaffold’s volume was 425.2 ± 46.8 mm^3^, which was comparable to the control side bone volume (489.2 ± 55.7 mm^3^). (Fig. 2A and Table 1).

**Table 1:** Distribution of the values of individual specimens retrieved using micro-computed tomography (CT) analysis for each group.

Specimen ID	A. Control side bone volume (mm^3^)	B. Examined side whole volume (mm^3^)	C. Remnant scaffold’s volume (mm^3^)	D. Regenerated bone volume (mm^3^)	E. Regenerated bone volume ratio (%)
**Group A (control)**					
18–2	331.5	322.0	307.7	14.3	4.3
18–3	589.3	491.8	404.9	86.9	14.8
18–4	536.6	559.0	530.1	28.9	5.4
18–17	499.5	477.7	458.3	19.3	3.9
Mean ± SEM	489.2 ± 55.7	462.6 ± 50.1	425.2 ± 46.8^a^	37.4 ± 16.8^a^	7.1 ± 2.6^a^
**Group B (rhBMP-2)**					
18–8	480.6	206.2	42.4	163.7	34.1
18–9	344.3	475.2	394.4	80.8	23.5
18–11	294.6	305.8	213.7	92.1	31.3
18–12	385.9	399.6	322.5	77.1	20.0
18–13	474.6	220.4	1.5	218.9	46.1
Mean ± SEM	396.0 ± 36.3	321.4 ± 51.7	194.9 ± 76.5^b^	126.5 ± 28.0^a^	31.0 ± 4.6^a^
**Group C (HMGB-1)**					
18–5	663.2	657.8	89.4	568.4	85.7
18–6	417.6	342.4	201.3	141.2	33.8
18–7	490.2	407.8	32.8	375.0	76.5
18–14	428.9	412.3	42.6	369.7	86.2
18–16	320.0	382.7	221.4	161.3	50.4
Mean ± SEM	464.0 ± 56.8	440.6 ± 55.7	117.5 ± 39.6^b^	323.1 ± 78.8^b^	66.5 ± 10.5^b^

Values are represented as mean ± SEM. Mean values within a column with unlike superscript letters were significantly different (*p* < 0.05), as determined using one-way ANOVA, followed by the Tukey–Kramer post-hoc test. Each value was calculated using the following equation: D = B – C, E = D/A × 100.

In group B (rhBMP-2), samples showed a regenerated bone volume of 126.5 ± 28.0 mm^3^ and the remnant scaffold’s volume was 194.9 ± 76.5 mm^3^ (Table 1). In most of the specimens, only minimal bone formation was noted at the base of the defect area, and a smaller amount of the scaffold material was present compared with the specimens in group A (control) (Fig. 2B).

In group C (HMGB-1), animals showed a regenerated bone volume of 323.1 ± 78.8 mm^3^ and the remnant scaffold’s volume was 117.5 ± 39.6 mm^3^ (Table 1). The regenerated bone formation was recognized as a structure surrounding the remnant scaffold material and bridging formation from the contralateral side. Morphological changes in the scaffold material were clearly identified by a decrease in radiodensity and volume (Fig. 2C).

**Figure 3:**
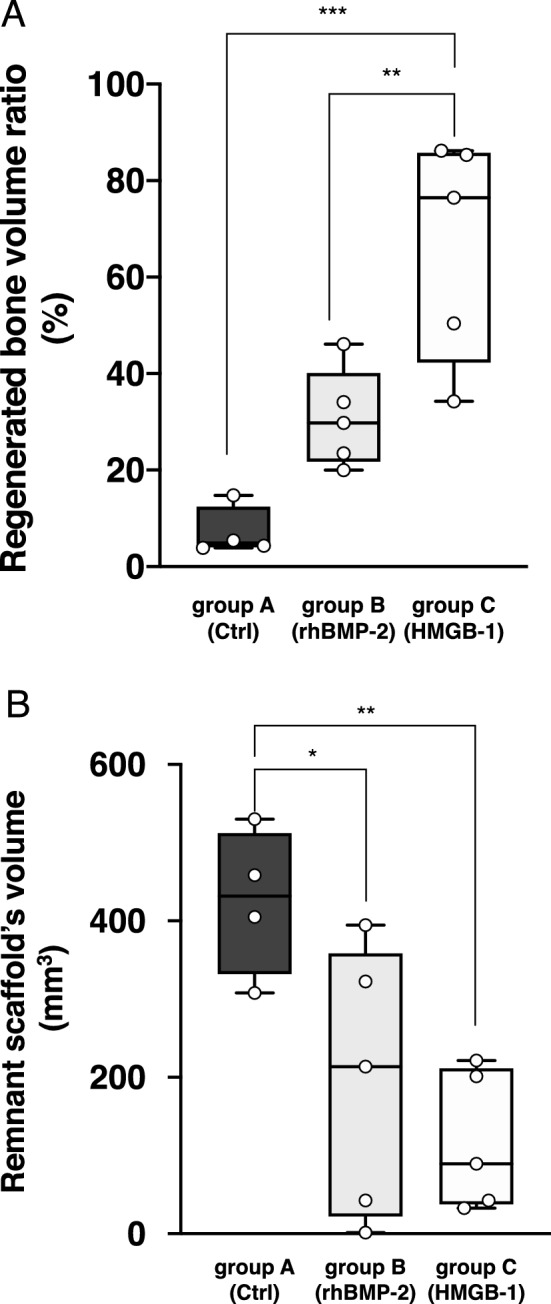
Comparison of the regenerated bone volume ratio and remnant scaffold volume among groups. The regenerated bone volume ratio among the three groups showed a negative correlation with the remnant scaffold’s volume. (**A**) The regenerated bone volume ratio in group C (HMGB-1) was significantly different from that in (control) and group B (rhBMP-2) (*p* = 0.0004 and 0.0097, respectively). The ratio was the highest in group C, followed by group B (hrBMP-2) and group A (control). (**B**) The remnant scaffold volume in group C (HMGB-1) and group B (rhBMP-2) showed a significant difference compared to group A (control) (*p* = 0.01 and 0.0481, respectively). The volume was highest in group A (control), followed by group B, and C. Statistical analysis was performed using one-way ANOVA, followed by the Tukey–Kramer post-hoc test; *p < 0.05; **p < 0.01; ***p < 0.001. PRISM 8 software ver. 8.3.0 (https://www.graphpad.com/scientific-software/prism/) was used for the analysis and graphing.

Further image evaluation using image processing software revealed the volume of new bone formation and the ratio of bone volume compared to the control side. Table 1 shows a comparison among the three groups in remnant scaffold volume, regenerated bone volume, and ratio of new bone formation. Statistical analysis revealed a highly significant difference between the HMGB-1 and control groups in the ratio of regenerated bone volume (*p* < 0.001), while a significant difference was also detected between the HMGB-1 and rhBMP-2 groups (*p* < 0.05) (Fig. 3A). The remnant volume of the scaffold material was then calculated. A negative correlation was found between the amount of regenerated bone and the residual scaffold material, where a higher amount of the remnant beta-tri-calcium phosphate (β-TCP) block was found in the control group (425.2 ± 46.8 mm^3^), while lower amounts were observed in the rhBMP-2 and HMGB-1 groups (194.9 ± 76.5 mm^3^ and 117.5 ± 39.6 mm^3^, respectively) (Fig. 3B, Table 1).

### Histological examination

**Figure 4:**
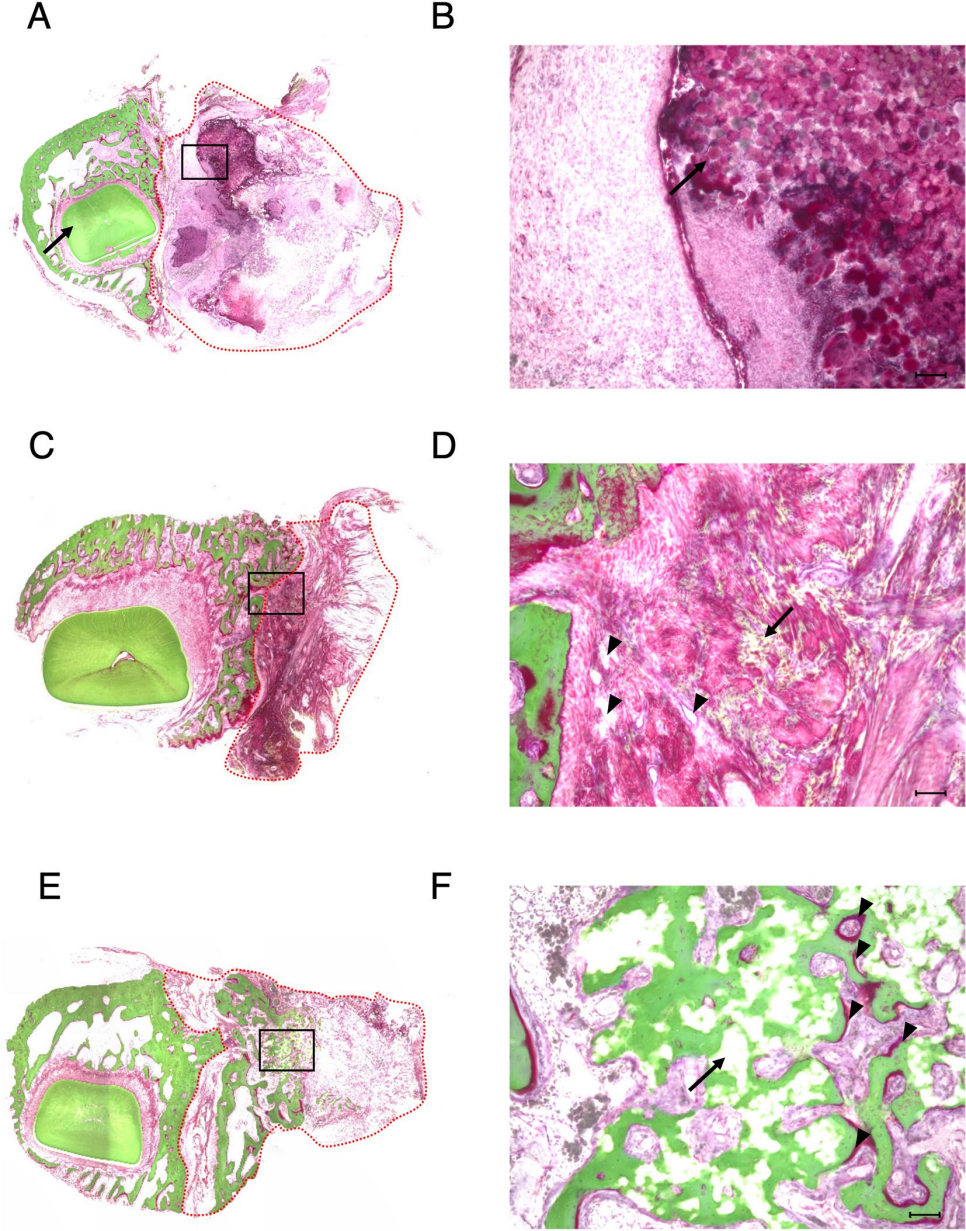
Histological examination with Villanueva–Goldner staining. (**A**) A representative histological image of group A (Control) is shown. The right-side mandible is designated as the control side, and the incisor root is identified (black arrow). (**B**) High magnification (10 ×) image from an enclosed rectangle area in (**A**). Remnant scaffold granules are predominant (black arrow), and little or no calcified structure is detectable. (**C**) In group B (rhBMP-2), no prominent callus formation is seen across the symphysis. (**D**) High magnification image from (**C**) shows blood vessels (black arrowheads), numerous fibroblasts, and scattered calcified tissue colored in green (black arrow). (**E**) In group C (HMGB-1), a callus formed across the symphysis. (**F**) High magnification image from (**E**) identified calcified bone surrounding the remnant scaffold material (black arrow). Osteoid (colored in red) is found adjacent to the calcified lamellar bone (black arrowheads). (**A**, **C**, **E**): magnification 2 ×; (**B**, **D**, **F**): magnification 10 ×. Scale bar = 100 μm. The red dotted line shows the area quantitatively assessed in Table 2.

**Figure 5:**
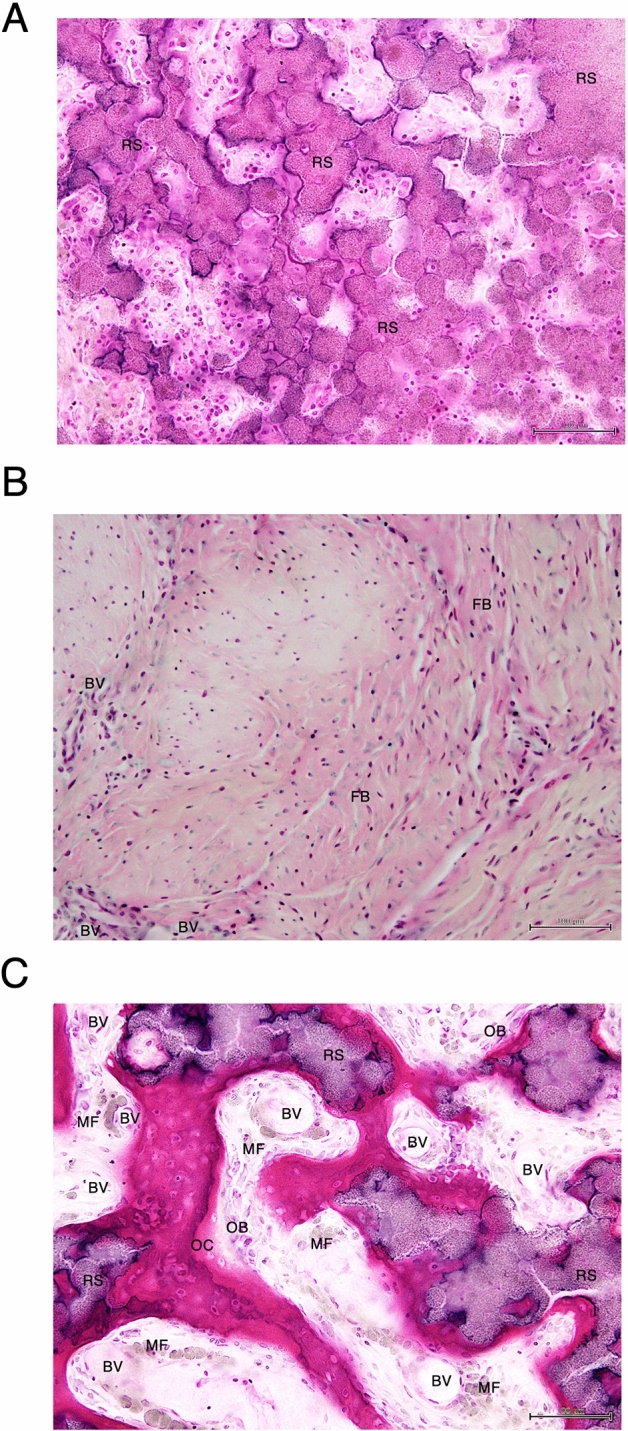
High magnification image of hematoxylin–eosin staining. The same specimens from Fig. 4 were investigated using hematoxylin–eosin staining at high magnification. (**A**) In the control specimen, the vast majority of the area was filled with remnant scaffold (RS). (**B**) The rhBMP-2 specimen shows abundant fibroblasts (FBs) with a small number of blood vessels (BV). (**C**) In the HMGB-1 specimen**,** RS is enclosed by a calcified bone structure. Active vascularization was confirmed by BVs. Macrophages (MF) phagocytizing β-TCP particles (grayish-green color) were observed along with the blood vessels. Numerous osteoblasts (OB) were identified on the osteoid surface, and osteocytes (OC) were encapsulated in the osteoid bone matrix. Magnification 20 ×. Scale bar = 100 μm.

The control specimen showed no osseous formation or covering for most of the defect side by the remnant β-TCP block (Fig. 4A,B) 12 weeks after the procedure. In the rhBMP-2 specimen, scattered calcified new bone formation was observed, represented by the green colored areas in Villanueva–Goldner staining. However, most of the defect side was filled with fibrous tissue, and the peripheral surface showed granulation tissue with blood vessels (Fig. 4C,D). Meanwhile, in the HMGB-1 specimen, woven bone was predominant, and an irregular structure with a high cellular component surrounding the remnant scaffold material was identified. Osteoids, represented in bright red, were recognized adjacent to the mature lamellar bone (Fig. 4E,F). Hematoxylin–eosin staining identified numerous osteoblast cells on the surface of the osteoid, and macrophages that phagocytize fine β-TCP particles around the blood vessels were also abundantly present in the HMGB-1 specimen (Fig. 5C). In contrast, remnant scaffold material without bone formation was predominant in the control specimen (Fig. 5A). Numerous fibroblasts without remnant scaffold material were detected in the rhBMP-2 specimen (Fig. 5B). Quantitative analysis of specimens using Villanueva–Goldner staining showed that group C (HMGB-1) had the highest bone volume ratio (BV/TV) of 24.23%, whereas group A (control) and group B (rhBMP-2) were 0.30% and 0.00%, respectively. Regarding the osteoid identified in bright red in Fig. 4F, the highest osteoid volume ratio (OV/TV) was observed in group C (HMGB-1) at 0.87%, followed by group A (Control) at 0.07%, and group B (rhBMP-2) at 0.00%. Conversely, the remnant scaffold volume ratio (RV/TV) was highest in group A (Control) at 32.66%, followed by group C (HMGB-1) at 12.83%, and group B (rhBMP-2) at 0.00% (Table 2).

**Table 2:** Quantitative analysis of histological tissue images with Villanueva–Goldner staining specimens of each group.

	Specimen ID	Total ROI volume (TV) (mm^2^)	Bone volume (BV) (mm^2^)	Osteoid volume (OV) (mm^2^)	Remnant Scaffold's volume (RV) (mm^2^)	Bone volume ratio BV/TV (%)	Osteoid volume ratio OV/TV (%)	Remnant Scaffold's volume ratio RV/TV (%)
Control	18–4	56.737	0.172	0.033	18.529	0.30	0.06	32.66
rhBMP-2	18–13	11.628	0.000	0.000	0.000	0.00	0.00	0.00
HMGB-1	18–14	33.856	8.203	0.290	0.345	24.23	0.87	12.83

Representative histological tissue images from each group were quantitatively analyzed within the region of interest (ROI) presented in Fig. 4A,C, and E.

## Discussion

In this study, we present two main findings. First, micro-CT examination showed a significantly higher regenerated bone volume ratio of 66.5% in group C (HMGB-1) than in group A (control) and group B (rhBMP-2), at 7.1% and 31.0%, respectively. Second, histological analysis of the specimens in group C (HMGB-1) revealed that the remnant scaffold material was enclosed by osteoid or newly formed mature bone, along with numerous osteoblasts on the surface of the osteoid and macrophages phagocytizing β-TCP particles.

Previous in vitro studies have suggested a direct effect of HMGB-1 on the proliferation and migration of osteoblasts^40^ and a critical role for HMGB-1 in bone fracture healing^39^. In agreement with the in vitro studies, the findings of this study showed the potential role of HMGB-1 as a sole bone-active molecule to facilitate the regeneration of critical-sized mandibular osseous defects in rabbits. To assess the healing process, a periodic X-ray examination was performed at four-week intervals to evaluate the volume of bone regeneration, tissue reaction, and the state of the scaffold material. In agreement with previous reports by Naudi et al. and Alfotawei et al.^14,42^, all groups showed a radiolucent line of demarcation between the radiodense scaffold and the mandibular bone at day 0, indicating a lack of calcified structure between the scaffold material and the surrounding bone structure.

The specimens in group C (HMGB-1) showed a change in the radiodensity of the scaffold material as early as four weeks after the procedure. Bridging between the scaffold material and the adjacent bone structure was observed, and the demarcation line had started to fade. At the end of the study (12 weeks), a large callus formation surrounding the remnants of the scaffold material was detected. These findings are consistent with a previous report by Xue et al., where they observed callus formation in rats treated with 10 µg of HMGB-1^41^. In contrast, the specimens in group B (rhBMP-2) did not show major structural changes until eight weeks postoperatively. The discrepancy in the time course of radiodensity may suggest the preferred efficiency of HMGB-1 compared with rhBMP-2 in the rapid restoration of critical-sized mandibular osseous defects. However, caution should be paid when interpreting the demarcation line in X-ray observations, since it only describes radiodensity under the conditions of this study, and does not necessarily indicate defects in bone structure.

The results from the micro-CT examination supported the X-ray examination, as group C (HMGB-1) showed the highest new bone regeneration ratio (66.5%) compared with the other two groups. These results are likely due to the direct effect of HMGB-1 on MSCs, which induces their osteogenic differentiation^37^, and its anabolic effect on osteoblast cells, which induces their proliferation and migration, which in turn improves bone restoration^40^. The highest regenerated bone volume ratio in group B (rhBMP-2) was 46.1%, which was approximately half of that in group C (HMGB-1). This finding is consistent with the report by Marukawa et al., in which they used rhBMP-2 with a collagen sponge and obtained a 56.3% defect filling in a segmental critical-sized mandibular defect in non-human primates; however, it should be noted that a higher dose (9 mg) of rhBMP-2 was used^27^. Because of the high solubility and short lifetime of rhBMP-2, previous preclinical in vivo studies have stated that a supra-physiological dose is required to achieve adequate bone healing for critical-sized mandibular defects^24,26,27,43^. The dose of rhBMP-2 used in this study was 10 µg/defect. Previous studies found that a dose of rhBMP-2 exceeding 20 µg/defect for critical size osseous defects was a supra-physiological concentration and more likely to induce several side effects, including cyst-like bone formation and soft tissue swelling^44,45^. In contrast, a dose between 2.5 and 10 μg was found to be safe and effective for various BMP release systems, including porous titanium^46^, alginate-based^47^, poly l-lactic acid (PLLA)-based^48^, and silk-based^49^ scaffolds. In agreement with these studies, no complications, including ectopic bone formation, were detected in group B (rhBMP-2). In group C, we used a dose of 10 μg/defect of HMGB-1 for direct comparison using the same factors as in group B (rhBMP-2). A recent study by Xue et al. demonstrated the ability of HMGB-1 gelatin sponge scaffolds to increase bone formation^41^. They applied a dose of 10 μg/defect of HMGB-1 in gelatin sponge scaffolds with mesenchymal stem cell sheets in a rat tibial osteotomy model. Although we recently showed that the maximum concentration of HMGB-1 was 150 μg/L for osteoblast viability in an in vitro study^50^, it remains to be elucidated whether 10 μg/defect of HMGB-1 is the maximum effective dose in vivo. In the present study, the volume of the remnant scaffold material was calculated using an image processing program. A negative correlation between the volume of the remnant scaffold material and the newly formed bone was found, suggesting that various cell types are involved in the degradation process by phagocytic mechanisms including monocytes/macrophages, fibroblasts, osteoblasts, and osteoclasts^51^. HMGB-1 has been shown to induce osteoblast proliferation and migration^40^, and to regulate the receptor activator of nuclear factor kappa B ligand (RANKL)-induced osteoclastogenesis in a receptor for advanced glycation end products (RAGE)-, TLR2-, TLR4-, and TLR9-dependent manner, which in turn, is involved in bone remodeling and maturation^52,53^. Consistent with this finding, the histological studies in group C (HMGB-1) revealed that the remnant scaffold material was enclosed by osteoid or newly formed bone, suggesting that the regenerated bone replaced the scaffold. In addition, numerous osteoblasts on the surface of the osteoid and macrophages phagocytizing β-TCP particles in hematoxylin and eosin staining indicate the involvement of these cells in the bone remodeling process. In contrast, the specimen in group B (rhBMP-2) was filled with numerous fibroblasts, but osteoblasts were hardly observed. Quantitative analysis of Villanueva–Goldner staining in the group C (HMGB-1) specimen showed excellent correlation with the micro-CT examination for evaluating bone volume, suggesting that the definition of the bone structure in micro-CT images was validated. It is important to note that bone tissue in the group B (rhBMP-2) specimen was not identified in the quantitative analysis, even though the regenerated bone volume was accounted for in the micro-CT analysis. This is because the histological specimen was a cross-sectional slice 10 mm anterior to the fixing screw where the section did not include the regenerated bone. The significant difference in the histological specimens between group B (rhBMP-2) and group C (HMGB-1) suggests that the mechanism of bone formation is different between the two molecules. However, the regenerated bone area at Hounsfield units between 1500 and 2000 was comparable among the groups, suggesting that the quality of formed cortical bones appear to be identical.

For the scaffold, β-TCP was used to control the shape of the regenerated bone, restoring the morphology and function of the excised bone segment. β-TCP has been studied extensively in the past decade as an alloplastic ceramic material^54,55^, and has been proven to have excellent osteoconductivity, biocompatibility, and resorbability when used for filling bone defects^54,56^. While scaffold materials with 70% and 80% porosity are commonly used in bone regeneration studies, the 60% β-TCP scaffold used in this study provides higher compressive strength and more mechanical support for the defect side. Tanaka et al. compared 75% and 60% porosity β-TCP blocks in opening wedge high tibial osteotomy^57^. They concluded that β-TCP with 75% porosity provided a low compressive strength of 3 MPa, which was inadequate for weight-bearing sites.

In contrast, 60% porosity provided 22 MPa compressive strength, but required longer a resorption time. The pore size of the β-TCP scaffold ranged from 300 to 500 µm in this study, representing the ideal size to support neo-vascularization 50 μm in diameter, which is a critical step in the bone healing process. Tricalcium phosphate implants mostly behave as osteoconductive materials, which permit bone growth on their surface or into pores, channels, or pipes^58^. Bashoor-Zadeh et al*.* reported an excellent correlation of β-TCP scaffold resorption between computational simulation data and experimental results in an in vivo sheep model^59^. They found a linear resorption rate of the scaffold until less than 5% ceramic remained at 24 weeks, as the pore size increased. A pore size of 510 μm showed significantly faster resorption rates compared to smaller pore sizes of 150 and 260 μm. However, in this study, group A (control) showed little, if any, β-TCP scaffold resorption at 12 weeks. This may be because the scaffold was installed in a rectangular defect where only two out of six walls were adjacent to the bone, suggesting that the defect type also contributes significantly to the resorption rate. Further examination is required to clarify whether different scaffold materials induce bone regeneration differently in combination with HMGB-1 or rhBMP-2.

To investigate the effect of HMGB-1 on the reconstruction of critical-sized osseous defects, we employed a rabbit mandibular defect model. The methodology was based on a previous report by Naudi et al. This model allows the creation of a large bone volume defect in the mandible consistently without compromising animal welfare, and is thus suitable for evaluating bone morphology using micro-CT analysis^42^.

The limitation of this study is the lack of previous studies examining the in vivo application of HMGB-1 for bone regeneration, and the need for further studies to compare and determine the optimal carrier system and concentration of HMGB-1. The mechanical properties of the regenerated bone were not measured, as we focused on the early stage of bone regeneration using HMGB-1. Long-term experiments are needed to assess the parameters in this defect model. In addition, a relatively small sample size was used in this study, so further experiments with larger sample numbers are required to demonstrate the feasibility of the clinical use of HMGB-1 as a sole bone-active molecule for the regeneration of critical-sized mandibular osseous defects. It should be noted that rhBMP-2 is clinically used combined with absorbable collagen sponge in general. Thus, the results of this study do not immediately apply to the preferred efficacy of HMGB-1 over rhBMP-2 in bone regeneration. In conclusion, this study demonstrated a preliminary outcome that an HMGB-1 loaded β-TCP scaffold can potentially become a novel practical method of intervention for the reconstruction of critical-sized mandibular defects.

## Methods

All experiments performed and reported here were in accordance with the ARRIVE guidelines^60^. The experiments were approved by the Animal Committee of Kyushu Dental University (approval number 18-015).

### Animals

The in vivo experiment was performed on 15 male New Zealand specific pathogen-free rabbits with an average weight of 2.5–3 kg and 5–6 months of age. The rabbits were maintained in the animal facility of Kyushu Dental University in a temperature-controlled room (22 °C) with ad libitum access to chow and water under a 12-h light/dark cycle. The animals were randomly assigned to three different groups in which the mandibular defect was filled with a β-TCP scaffold mounted with phosphate-buffered saline (PBS) (group A; n = 4), rhBMP-2 (group B; n = 5), and HMGB-1 (group C; n = 5). All groups originally contained five animals, but one sample in the control group was excluded from the assessment due to complications during sample processing.

### Scaffold material

The scaffold material used in this study was β-TCP (Brain Base Co., Ltd. Tokyo, Japan). All test scaffolds were sterilized using gamma irradiation at a maximum of 40 kGy before surgical intervention. The porosity was 60%, and the average pore size ranged from 400 to 500 µm. The 20 × 10 × 10 mm β-TCP scaffold block was trimmed to fit the actual shape of the defect at the time of surgery.

### Recombinant human BMP-2 (rhBMP-2)

Highly purified, freeze-dried recombinant human BMP-2 (Novus Biologicals, Littleton, CO, USA) produced in a Chinese hamster ovary cell line with a purity greater than 90% was used in this experiment. rhBMP-2 was supplied in a plastic vial containing 10 µg of lyophilized rhBMP-2, aliquoted, and stored at − 28 °C until use.

### Recombinant human HMGB-1

Highly purified, freeze-dried recombinant human HMGB-1 (R&D Systems, Minneapolis MN, USA) produced in a mouse melanoma cell line culture with 95% purity was used. HMGB-1 was supplied in a glass vial containing 50 µg lyophilized HMGB-1 and stored at – 28 °C until use. At the time of surgery, lyophilized HMGB-1 was reconstituted in sterile PBS according to the manufacturer’s instructions.

### Surgical intervention

#### Preoperative preparation

Each animal was subcutaneously pre-medicated 1 h before surgery with the nonsteroidal anti-inflammatory drug (NSAID) meloxicam (0.2 mg/kg) and a prophylactic dose of the antibiotic oxytetracycline (30 mg/kg). Animals were anesthetized by administering ketamine (35 mg/kg), xylazine (5 mg/kg), and butorphanol (0.1 mg/kg) via intramuscular injection.

#### Surgical procedure

In all animals, the left mandible was assigned to the surgical intervention side. A linear incision was made along the inferior border of the mandibular body on the left side, and the buccinator muscle was elevated. After exposure of the left mandibular ramus, the mental foramen and mental nerve were identified, and the mental neurovascular bundle was ligated and cut.

The integrity of the mandible was maintained before creating the continuity defect by insertion of a 2.0 mm bi-cortical self-tapping, 13-mm-long titanium screw (KLS Martin, Tuttlingen, Germany) through the body of the mandible from one side to the contralateral side, just anterior to the first premolar tooth. An X-ray image was taken to ensure that the screw was in place and reached the other side of the mandible (day 0).

**Figure 6:**
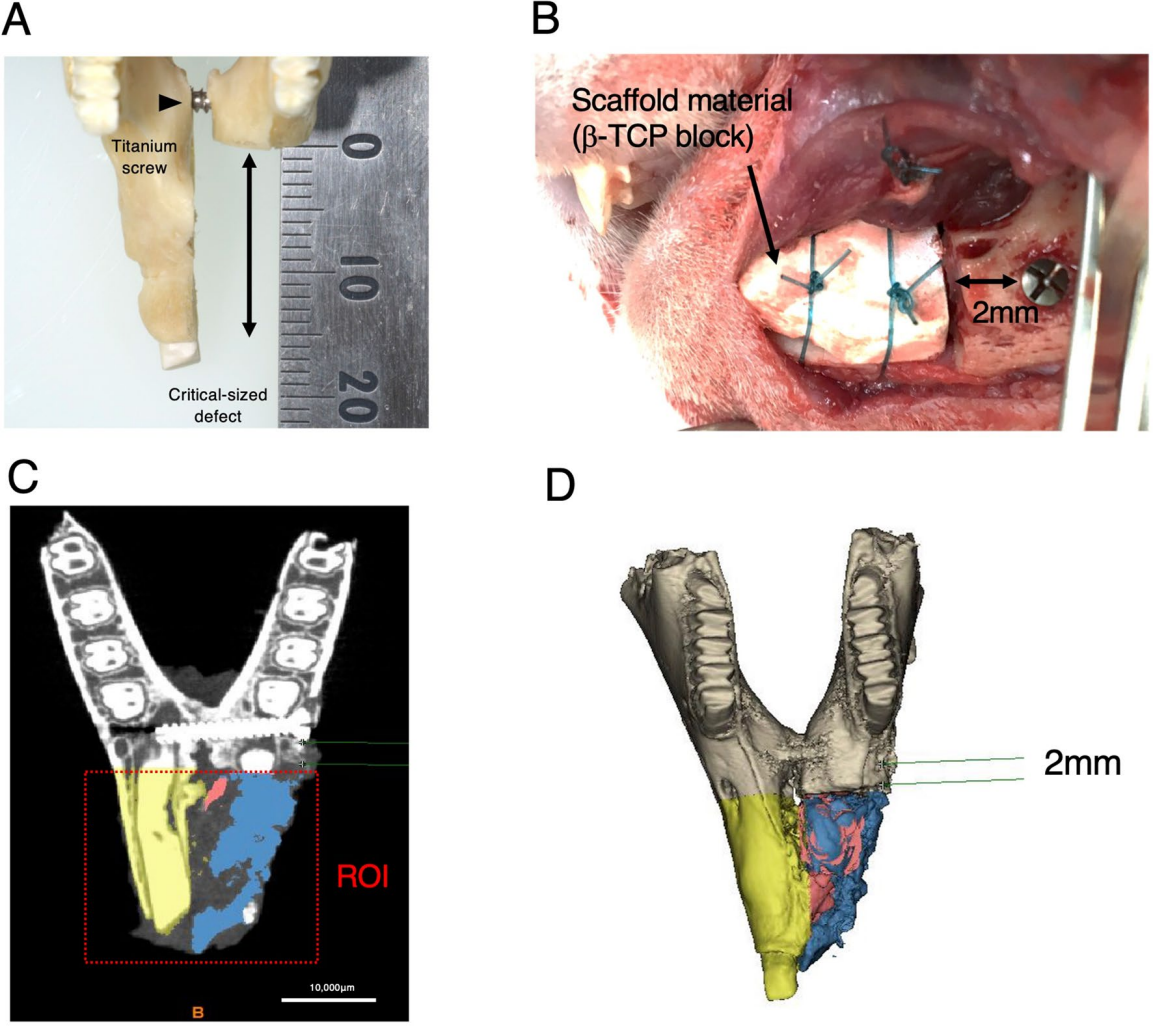
Surgical procedure for installing the scaffold material on the critical-sized osseous defect. (**A**) The approximately 15 mm long critical-sized osseous defect was created on the left side rabbit mandible (examined side). (**B**) Scaffold material (β-TCP block) was installed on the osseous defect and secured with absorbable sutures. (**C**) The region of interest (ROI) was defined as the anterior part of the mandible separated by the horizontal line 2 mm anterior to the medial edge of the fixing screw. In every cross-sectional slice of the micro-CT image, the images were split into the control side (yellow) and the examined side using an image processing software. The examined side was then separated into bone (blue) and remnant scaffold (red) based on its Hounsfield unit. (**D**) Reconstructed 3-D CT image retrieved from (**C**).

The length of the removed segment was determined using a surgical caliber to create an approximately 15 mm bony defect. Straight body osteotomy was then performed by making two straight cuts using a saw cutting rotary burr, where the first cut was made 2 mm anteriorly to the first premolar, right in front of the fixing screw sectioning the mental nerve, and the second cut was performed across the midline, in the region of the mandibular symphysis (Fig. 6A).

The bone segment was removed with the associated periosteum and incisor teeth, creating a 15-mm-long critical-sized defect. The β-TCP block was trimmed to fit the defect using the segment of the bone that had been removed (Fig. 6B). The scaffold was then placed in a sterilized dish, and test samples were prepared as follows.

For group A, 100 µL of sterile PBS was loaded in drops onto the β-TCP block, and then translocated to the surgical site. For group B, lyophilized rhBMP-2 was reconstituted according to the manufacturer’s specifications in 100 µL of 4 mM HCl, and the whole 100 µL was loaded onto the β-TCP block in the same manner (10 µg of rhBMP-2/scaffold). For group C, lyophilized HMGB-1 (50 µg) was reconstituted in PBS (0.1 µg/µL), then 100 µL was loaded onto the β-TCP scaffold using a micropipette in drops, until it was entirely immersed (10 µg of HMGB-1/scaffold). The surgical procedure was performed as previously described^42^. Briefly, the β-TCP scaffold prepared in each group was fixed with the contralateral half of the mandible using two simple interrupted sutures surrounding the scaffold with absorbable suture material (MAXON 3/0, Medtronic, Dublin, Ireland) (Fig. 6B). The anterior intraoral mucosal defect created by removing the left incisor was sutured using a non-absorbable suture material (NYLON 5/0, Natsume Seisakusho, Tokyo, Japan) using one or two interrupted suture patterns.

The scaffold was then secured in the surgical defect by enclosing it within the surrounding soft tissues, and an X-ray examination was performed to ensure that the scaffold material was secured in the correct position.

#### Post-operative care

A successive dose of NSAIDs followed by meloxicam (0.2 mg/kg) was administered postoperatively for two days, while the antibiotic oxytetracycline (30 mg/kg) was administered only on the day of surgery. Daily examination of the surgical site was conducted for swelling, abscess formation, and wound dehiscence. The skin suture was removed 10 days postoperatively. The food was converted from hard pellets to soft powder at this time, and continued for the rest of the study.

#### Radiographic examination (X-ray)

A digital X-ray system (NAOMI, Nagano, Japan) was employed for periodic examination of the anterior portion of the mandibular ramus. The X-ray was laterally directed to an imaging plate equipped with a PC. Examinations were performed immediately after the surgery (Day 0), followed by 4, 8, and 12 weeks postoperatively.

#### Micro-CT examination

All cases were euthanized at 12 weeks after the procedure with an overdose (140 mg/kg) of pentobarbital sodium (Kyoritsu Pharmaceutical, Tokyo, Japan) administered intravenously. The mandibles were surgically removed and stored in 10% neutral buffered formalin (FUJIFILM Wako Pure Chemical Corporation, Osaka, Japan) at 4 °C for five days, and preserved in 70% ethanol for further micro-computed tomography (CT) and histological examination.

All samples were shipped to the Kureha Analysis Center (KSL, Fukushima, Japan) for micro-CT examination. Samples were analyzed using Scan XMATE-L090H (Comscan Techno, Kanagawa, Japan) with the following parameters: voltage, 80 kV; current, 100 μA; magnification, 2.4807 ×; resolution, 102.392 μm/pixel; slice thickness, 102.392 μm. The region of interest (ROI) assigned for the CT examination was as follows:

In the axial slice image, the starting point was defined as 2 mm anterior to the most medial edge of the screw. From this point, a horizontal line parallel to the fixing screw was drawn, and the whole anterior section separated by the line was defined as the ROI (Fig. 2). CT digital imaging and communications in medicine (DICOM) data were converted and reconstructed to 3-D images using an image processing and analyzing program (Materialise MIMICS, Materialise Technology, Leuven, Belgium), and then the regenerated bone volume of the examined side (left side) was retrieved in each group by removing the remnant scaffold volume from the entire examined side volume.

Using the split mask option in the program, the control side (yellow) was manually separated from the defect side (red), and then the remnant scaffold material was identified based on its Hounsfield unit, where the threshold well over 2000 was isolated with support of manual segmentation by an investigator blinded to this study on the defect side (blue) (Fig. 6C,D), and then the 3D images were constructed and analyzed (Table 1). The baseline threshold of Hounsfield unit was determined by taking micro-CT of β-TCP scaffold before installation in the defect (Supplementary Fig. S1). To standardize the regenerated bone volume, the regenerated bone volume ratio was calculated as follows:

$${\text{Regenerated }}\;{\text{bone}}\;\;{\text{ volume}}\;{\text{ ratio }}\left( \% \right) = \left[ {{\text{examined }}\;{\text{side }}\;{\text{whole }}\;{\text{volume }}\left( {{\text{mm}}^{3} } \right){-}{\text{Remnant }}\;{\text{Scaffold's }}\;{\text{volume }}\left( {{\text{mm}}^{3} } \right)} \right]/{\text{control }}\;{\text{side}}\;{\text{ bone}}\;{\text{ volume }}\left( {{\text{mm}}^{3} } \right) \times 100.$$

The Hounsfield unit between 1500 to 2000 was segmented and defined as hard cortical bone within the regenerated bone to assess bone quality (Supplementary Table S1).

#### Histological examination

Following micro-CT examination, the samples were subsequently fixed and stained as previously described^61^. Briefly, the samples were embedded in methyl methacrylate, and the undecalcified ground specimens were cross-sectioned into 30 μm slices using a microtome (Exakt Technologies, Norderstedt, Germany) at a point located 10 mm anterior to the edge of the fixing screw. Villanueva–Goldner staining was performed to identify mineralized bone and osteoid formation. Hematoxylin–eosin staining was performed to examine the specimens at the cellular level. All histological images were captured using BZ-9000 (Keyence Japan, Osaka, Japan). Quantitative analysis was performed with Villanueva–Goldner staining using the Histometry RT CAMERA system (System Supply, Tokyo, Japan) at 40 × magnification (Table 2).

### Statistical analysis

All data were analyzed using one-way ANOVA, followed by the Tukey–Kramer post-hoc test for multiple comparisons (PRISM 8 software ver. 8.3.0, GraphPad Software, San Diego, CA, USA). Statistical significance was set at *P* < 0.05. The equality of variance was ensured by performing the Brown–Forsythe test.

## Supplementary Information

Supplementary Information.

## Data Availability

The data that support the findings of this study are available from the corresponding author upon reasonable request.
