# Supplementary material for: Local administration of HMGB-1 promotes bone regeneration on the critical-sized mandibular defects in rabbits
Source: Sci Rep. 2021 Apr 26;11:8950. doi: 10.1038/s41598-021-88195-7 (PMC8076241; doi:10.1038/s41598-021-88195-7)
Supplement: Supplementary file 1 — Supplementary Information. [file 41598_2021_88195_MOESM1_ESM.docx]

**Local administration of HMGB-1 promotes bone regeneration on the critical-sized mandibular defects in rabbits**

**Ahmed Monir, Taro Mukaibo, Abdel Basit M. Abd El-Aal, Tomotaka Nodai, Takashi Munemasa, Yusuke Kondo, Chihiro Masaki, Mahasen A. El- Shair, Kou Matsuo, and Ryuji Hosokawa**

**Supplementary Figure 1 Micro-CT assessment of β-TCP scaffold block**

Hounsfield unit at 2000< identified β-TCP scaffold block entirely.

The unit was referred to as the baseline to determine the Hounsfield unit's threshold to support identifying the remnant scaffold in animals' mandibular defect.


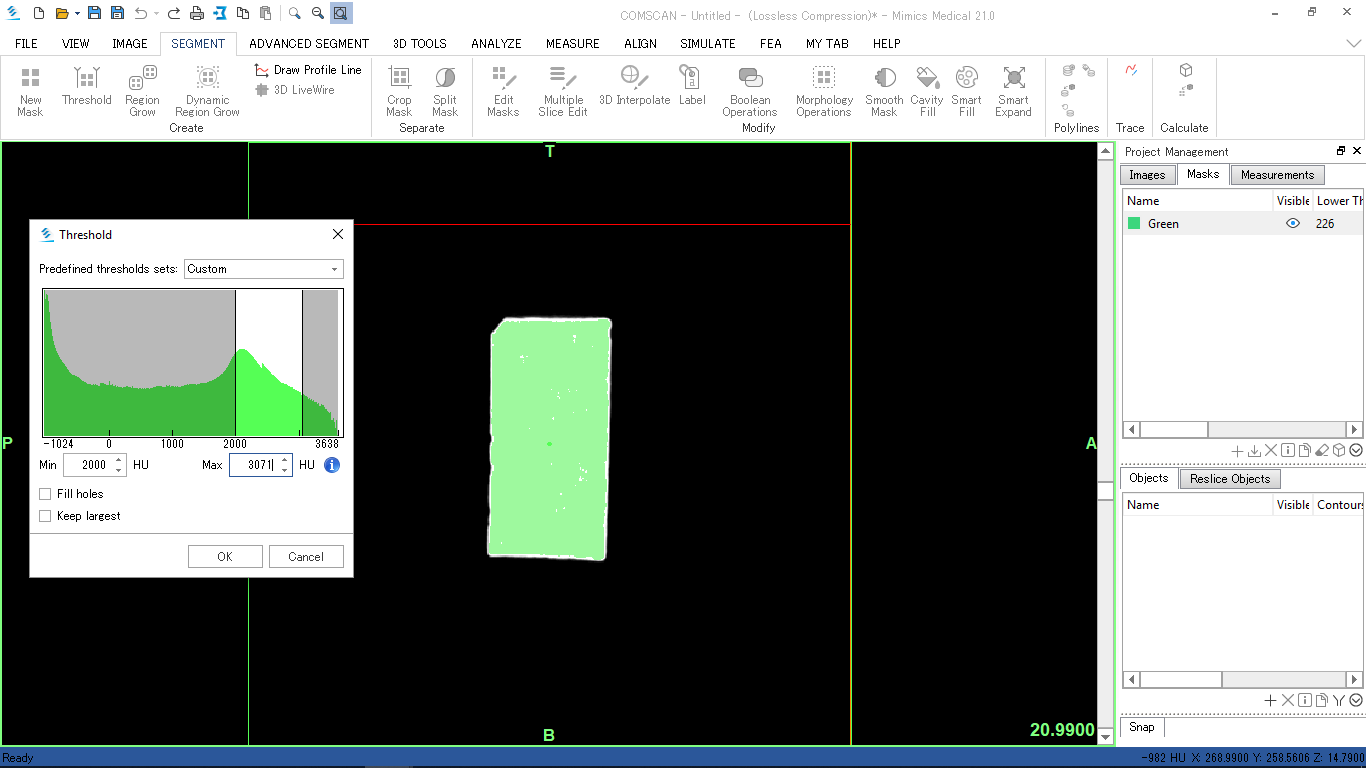


**Supplementary Figure 2 Individual axial micro-CT images among groups**

Axial micro-CT images of each specimen are shown.

**Group A (Control) Group B (rhBMP-2) Group C (HMGB-1)**

**18-2 18-8 18-5**

**
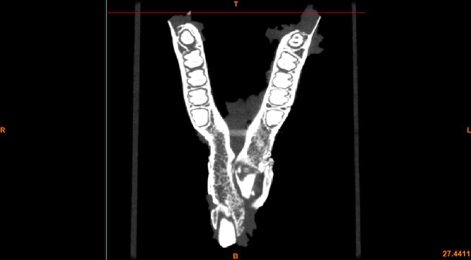

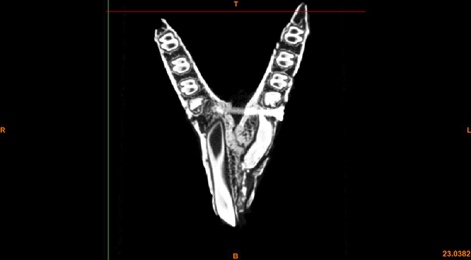

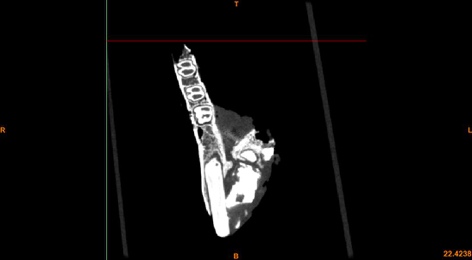
**

**18-3 18-9 18-6**

**
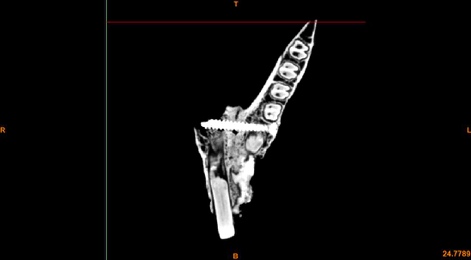

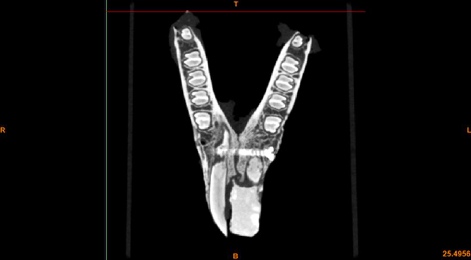

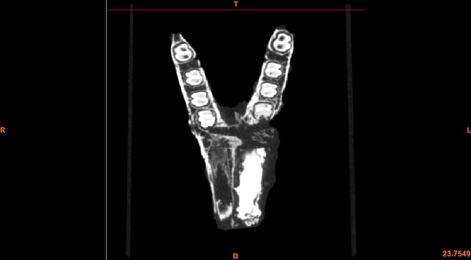
**

**18-4 18-11 18-7**

**
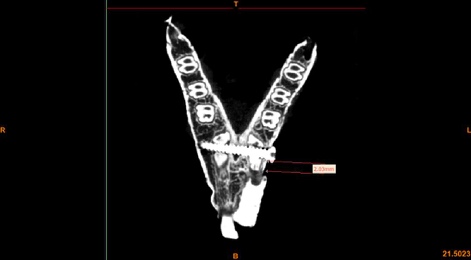

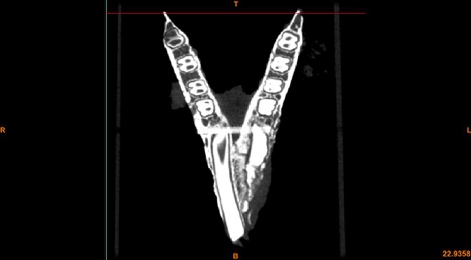

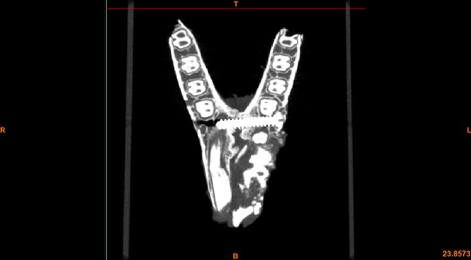
**

**18-17 18-12 18-14**

**
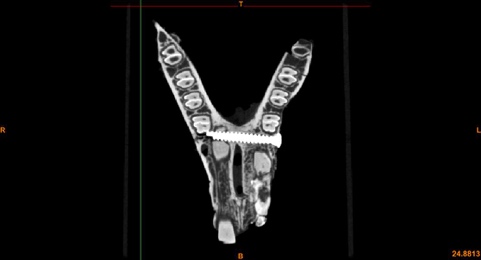

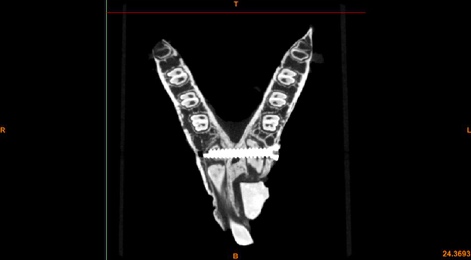

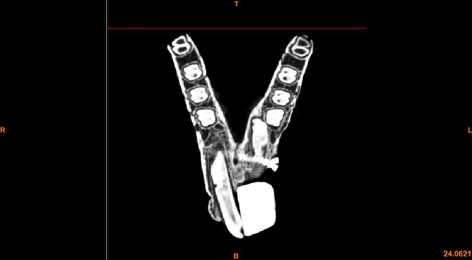
**

**18-13 18-16**

**
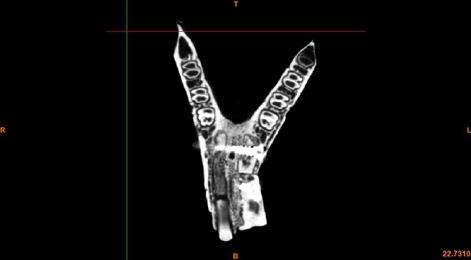

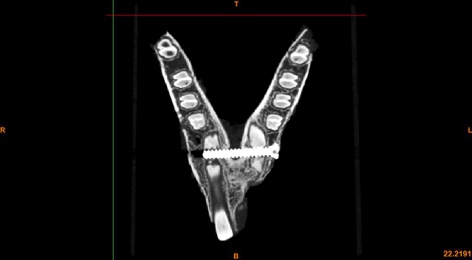
**

|  | Specimen ID | 1. Regenerated bone volume  (mm^3^) | 1. Bone volume at Hounsfield unit 1500-2000   (mm^3^) | 1. Bone volume ratio at Hounsfield unit 1500-2000   (%) |
| --- | --- | --- | --- | --- |
| **group A (Control)** |  |  |  |  |
|  | 18-2 | 14.3 | 1.7 | 11.6 |
|  | 18-3 | 86.9 | 21.6 | 24.8 |
|  | 18-4 | 28.9 | 3.3 | 11.5 |
|  | 18-17 | 19.3 | 4.8 | 24.6 |
| Mean± SEM |  | **37.4 ± 16.8^a^** | **7.8 ± 8.2^a^** | **18.1 ± 3.8** |
| **group B (rhBMP-2)** |  |  |  |  |
|  | 18-8 | 163.7 | 56.0 | 34.2 |
|  | 18-9 | 80.8 | 17.3 | 21.4 |
|  | 18-11 | 92.1 | 11.6 | 12.6 |
|  | 18-12 | 77.1 | 15.0 | 19.4 |
|  | 18-13 | 218.9 | 31.1 | 14.2 |
| Mean± SEM |  | **126.5 ± 28.0^a^** | **26.2 ± 8.2^a^** | **20.4 ± 3.8** |
| **group C (HMGB-1)** |  |  |  |  |
|  | 18-5 | 568.4 | 104.6 | 18.4 |
|  | 18-6 | 141.2 | 38.1 | 27.0 |
|  | 18-7 | 375.0 | 61.4 | 16.4 |
|  | 18-14 | 369.7 | 67.8 | 18.3 |
|  | 18-16 | 161.3 | 42.8 | 26.5 |
| Mean± SEM |  | **323.1 ± 78.8^b^** | **62.9 ± 11.8^b^** | **21.3 ± 2.3** |

**Supplementary Table 1 quality assessment of regenerated bone segmented at Hounsfield unit 1500-1400**

Values are represented as mean ± SEM. Mean values within a column with unlike superscript letters were significantly different (*p* < 0.05), as determined using one-way ANOVA, followed by the Tukey–Kramer post-hoc test. The following equation was used to calculate the Bone volume ratio at Hounsfield unit 1500-2000: C=B/A × 100
